# Research on the Axial Compression Performance of Double C-Section Partially Encased Composite Columns

**DOI:** 10.3390/ma19101931

**Published:** 2026-05-08

**Authors:** Ming Zhou, Linglin Qin, Xiaodong Wen, Feifan Wang, Gongwei Weng

**Affiliations:** 1 School of Architecture and Transportation Engineering, Ningbo University of Technology, Ningbo 315211, China; 2 Ningbo Construction Engineering Group Co., Ltd., Ningbo 315000, China

**Keywords:** cold-formed thin-walled steel, lightweight aggregate concrete, composite column, axial compression test, finite element analysis

## Abstract

To investigate the axial compressive behavior of double C-section partially encased composite (DCPEC) columns, 10 DCPEC specimens and two back-to-back bare steel C-section specimens were designed and tested under axial compression. The effects of key parameters, including steel wall thickness, member slenderness ratio, connection type of the built-up double C-sections and connection density, on the failure mode, load–displacement response and ultimate load-carrying capacity were examined. The test results showed that, under otherwise identical conditions, the ultimate load of the bolted stub column was 8.4% higher than that of the welded stub column. When the steel wall thickness increased from 2.0 mm to 3.0 mm, the ultimate load increased by approximately 16%. In contrast, when the slenderness ratio increased from 25.98 to 41.57, the ultimate load decreased by approximately 30%. A finite element model was then established in ABAQUS and validated against the experimental results. The numerical analysis further confirmed that increasing the slenderness ratio reduced the axial load-carrying capacity, whereas increasing the steel wall thickness improved the resistance of the member. The results indicate that the proposed DCPEC column can effectively develop the composite action between cold-formed thin-walled steel and lightweight aggregate concrete, thereby improving axial resistance and showing promising potential for engineering applications.

## 1. Introduction

Cold-formed steel (CFS), as a lightweight and environmentally friendly structural material, has been widely used in building structures owing to its low self-weight and high stiffness [1], particularly in light-gauge residential systems in which CFS serves as the main structural framework [2]. However, CFS members commonly adopt monosymmetric open sections, which generally exhibit limited torsional resistance and significant differences between the moments of inertia about the two principal axes, making them susceptible to instability. To improve the stability performance of single-limbed open-section members, individual CFS sections are often assembled with screws to form biaxially symmetric built-up I-sections [3], which have been widely applied in compression members such as struts in steel trusses and space frames, wall studs in framing systems and columns in portal frames. Despite these advantages, including light weight, high strength and ease of construction, steel itself has relatively poor corrosion and fire resistance and CFS members are sensitive to initial geometric imperfections, which to some extent restricts the wider engineering application of CFS columns. To overcome these limitations, based on the structural concept of partially encased composite (PEC) columns, cold-formed steel sections can be combined with concrete to form CFS partially encased composite columns, thereby reducing imperfection sensitivity and enhancing the load-carrying capacity of the members.

Therefore, the Double C-section partially encased composite column (DCPEC column) can fully exploit the respective performance advantages of cold-formed steel (CFS) and lightweight aggregate concrete (LWAC) [4]. It can effectively utilize the supporting role of CFS as permanent formwork for concrete, thereby reducing formwork demand and improving construction efficiency; meanwhile, during service, the confinement provided by CFS can enhance the contribution of lightweight aggregate concrete to the load-carrying capacity of the member [5,6]. In addition, the concrete infilled between the flanges can prevent local buckling of the web and flanges, enhance the lateral stiffness of the member, improve its seismic ductility and improve the corrosion resistance, fire resistance and thermal insulation performance of CFS [7]. Owing to these advantages, DCPEC columns exhibit broad application prospects and can be used not only for the strengthening and retrofitting of steel columns in existing light steel structures, but also directly in new structures.

In recent years, substantial progress has been made in the study of the axial compressive capacity of steel–concrete composite PEC columns. Existing studies have shown that the load-carrying capacity and failure characteristics of such members are significantly affected by factors including section configuration, concrete type and constructional parameters. Bangprasit [8] pointed out that the slenderness ratio has a significant influence on the load-carrying capacity of H-shaped steel PEC stub columns. Lai BL [9] proposed a simplified calculation formula for high-strength concrete PEC stub columns that takes into account the effect of increased concrete strength. Xu Mingke [10] found that the ultimate failure mode of cruciform PEC columns is basically similar to that of conventional PEC columns. Subsequent studies further extended to different concrete types and cold-formed thin-walled members. Pereira MF [11] found that the use of steel fiber-reinforced concrete did not significantly reduce the load-carrying capacity or mechanical performance of PEC columns. Simin Jian [12] showed that the spacing of transverse battens affects the ductility of recycled aggregate concrete PEC columns. Huo XY [13] pointed out that the load-carrying capacity of concrete-filled cold-formed thin-walled C-section steel stub columns increases with an increase in the sectional dimensions of the C-section steel. With regard to lightweight aggregate concrete composite members, Li [14] investigated the buckling behavior and failure modes of cold-formed steel–lightweight aggregate concrete composite columns under axial compression and analyzed the effects of steel plate thickness and concrete strength. Bonopera [15] verified the load-carrying capacity and engineering applicability of lightweight aggregate concrete-filled steel tubular columns through axial compression tests. Aleliwi B [16] showed that a proper lightweight aggregate concrete mix proportion can improve the overall load-carrying capacity of lightweight aggregate concrete-filled cold-formed C-section steel beams. Li Y [17] pointed out that increasing concrete strength can not only enhance the load-carrying capacity of lightweight aggregate concrete-filled steel tubular columns but also suppress buckling of the steel tube wall.

On the basis of experimental investigations, corresponding methods for predicting load-carrying capacity have also been developed. Based on the Eurocode and in combination with finite element analysis and the effective width method, Alabedi [18] improved the calculation formula for the load-carrying capacity of composite members composed of polystyrene lightweight aggregate concrete and cold-formed steel. Qian [19] further proposed modification coefficients to account for flange strength reduction and the contribution of the core concrete and verified their applicability. Overall, previous studies have provided an important basis for the mechanical analysis and load-carrying capacity prediction of PEC columns. However, existing research has still mainly focused on conventional welded H-shaped steel or cruciform sections, whereas studies on PEC columns composed of cold-formed C-section steel and other special-shaped sections remain limited and the corresponding mechanical behavior and design methods have not yet been systematically established. Meanwhile, investigations into the combination of lightweight aggregate concrete and PEC columns are also relatively scarce and a comprehensive understanding of their mechanical performance, failure modes and long-term durability is still lacking. Therefore, research on double C-section partially encased composite columns (DCPEC columns) is of clear significance. This composite form not only helps reduce structural self-weight while improving load-carrying capacity but also allows lightweight aggregates to be prepared from industrial solid waste, thereby meeting the requirements of green construction and sustainable development. In addition, previous studies have shown that lightweight aggregate concrete infill can enhance the load-carrying capacity of members and suppress distortional buckling, which further demonstrates the necessity of investigating DCPEC columns.

In summary, previous studies on PEC columns have mainly focused on conventional welded H-shaped or cruciform sections, whereas research on cold-formed thin-walled C-section partially encased lightweight aggregate concrete composite columns remains limited. In particular, a systematic understanding is still lacking with respect to the axial compressive behavior, the influence of key parameters and the interaction mechanism between cold-formed thin-walled steel and lightweight aggregate concrete. To address these gaps, this study proposes a double C-section partially encased composite column (DCPEC column) and experimentally investigates the effects of connection type, steel wall thickness, member slenderness ratio and connection density on the failure mode, load–displacement response and ultimate load-carrying capacity of the member through tests on 10 DCPEC specimens and 2 bare steel reference specimens. On this basis, an ABAQUS finite element model is established and validated and a parametric analysis is further carried out to reveal the influence of slenderness ratio and steel wall thickness on the axial compressive performance of the member. The study is expected to provide a useful reference for the mechanical analysis, design optimization and engineering application of DCPEC columns.

## 2. Test Overview

### 2.1. Specimen Design and Fabrication

**Figure 1:**
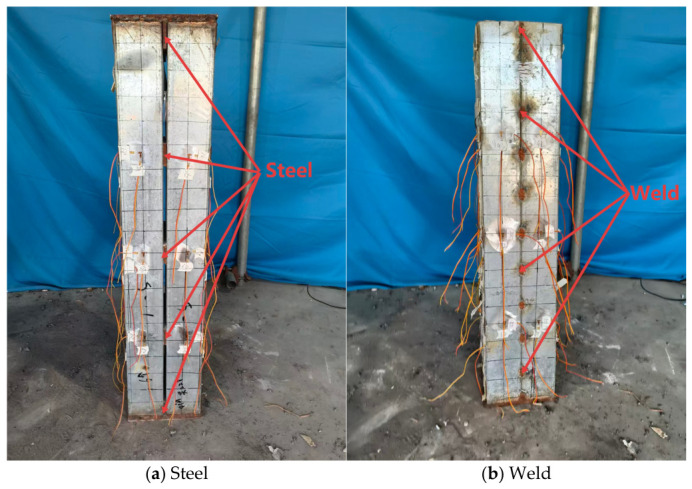
Schematic diagram of steel plate connections and weld points in composite members.

**Figure 2:**
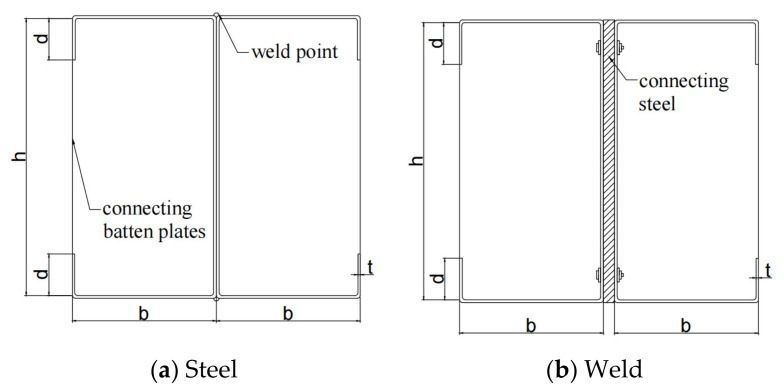
Section of specimens.

**Figure 3:**
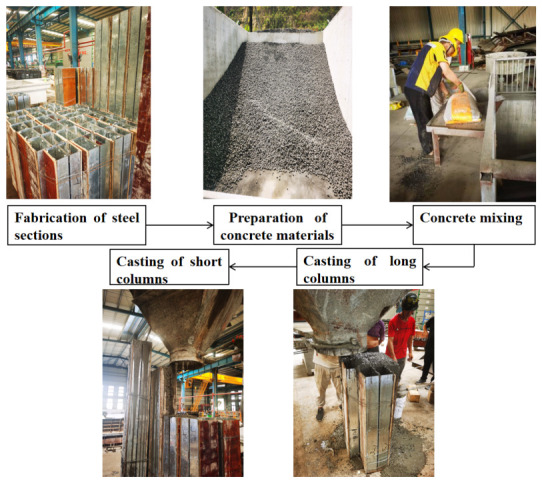
Technological flow sheet.

In this study, 10 double C-section partially encased composite (DCPEC) columns and 2 bare built-up steel columns were designed and fabricated. According to the connection type, the specimens were divided into bolted built-up sections and welded built-up sections, as shown in Figure 1. The detailed specimen parameters are listed in Table 1 and the sectional configuration is shown in Figure 2. The specimen heights were 1000 mm, 1500 mm, 2000 mm and 2400 mm. To prevent separation between the lips and the internal concrete, a 1 mm thick steel plate was welded to the lip of each C-section steel member, with a weld length of 50 mm at the upper and lower ends and 30 mm in the middle region. A connecting steel plate measuring 200 mm × 50 mm × 8 mm was arranged between the two C-section steels and connected using ordinary Grade 5.6 bolts, with a bolt edge distance of 50 mm. To facilitate concrete casting, a sealing plate measuring 215 mm × 205 mm × 8 mm was provided at the column base. The steel members were fabricated by Zhejiang Hengyun Metal Components Co., Ltd. (Ningbo, China). Both ends of the specimens were designed as pin-ended supports and realized using knife-edge hinge supports. End plates measuring 377 mm × 307 mm × 30 mm were installed at both ends to ensure reliable transfer of load to the supports. During fabrication, the built-up double-limb C-section steel skeleton was first assembled, after which the lightweight aggregate concrete was prepared and cast. The fabrication procedure is shown in Figure 3.

To systematically investigate the axial compressive behavior of DCPEC columns, in addition to fabricating composite members, this study also specifically designed and fabricated pure steel double C-section columns for comparative tests. The main purpose was to obtain the load-carrying capacity and failure modes of the pure steel members under the same geometric dimensions, steel strength, boundary conditions and loading protocol, so that the contribution of the lightweight aggregate concrete infill to the enhancement of member load-carrying capacity could be quantitatively separated on this basis. At the same time, the typical failure modes of the pure steel columns can serve as an important reference for analyzing the restraining effect of concrete on the local stability of the steel sections in the composite columns and for verifying whether the concrete effectively delays or suppresses the premature buckling of the C-section steel.

### 2.2. Material Properties and Mix Proportion

**Figure 4:**
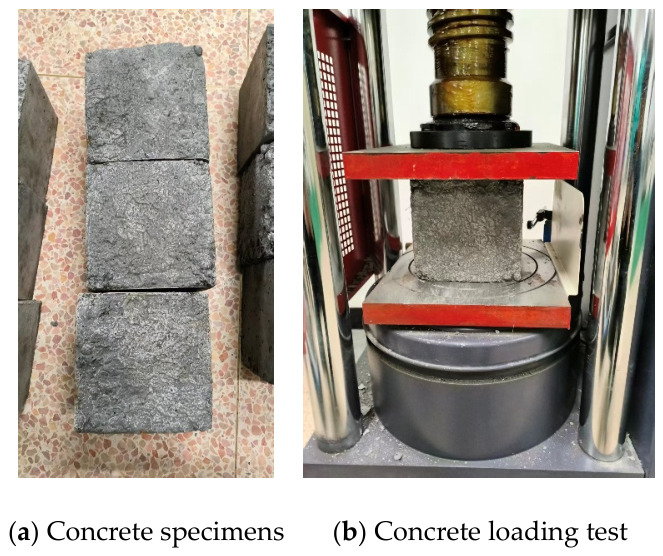
Compressive strength test of concrete.

**Figure 5:**
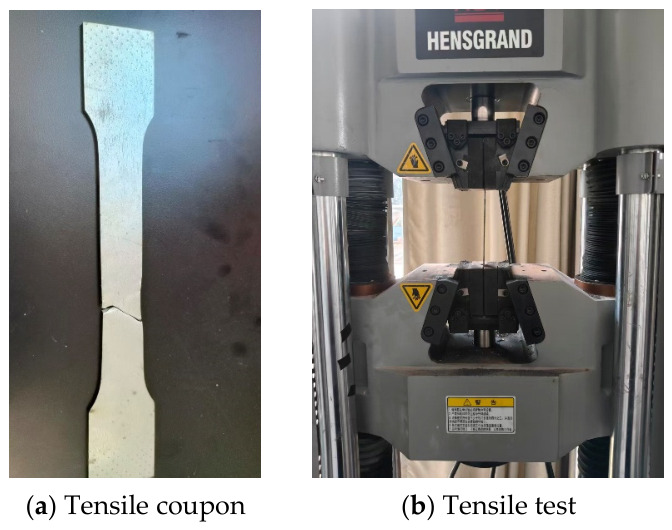
Mechanical properties test of steel.

Each specimen consisted of double C-section cold-formed thin-walled steel and lightweight aggregate ceramsite concrete. The lightweight aggregate concrete was designed with a strength grade of LC25. The ceramsite used in this study was fly ash ceramsite supplied by Ningbo Pinghai Building Materials Co., Ltd (Ningbo, China). The corresponding mix proportion is presented in Table 2 and the measured compressive strength was 26.8 MPa (details of the test specimens and the experimental procedure are shown in Figure 4). The cold-formed thin-walled steel was made of Q195 steel and its material properties are listed in Table 3 (details of the test specimens and the experimental procedure are shown in Figure 5).

### 2.3. Experimental Loading Setup and Instrumentation Layout

**Figure 6:**
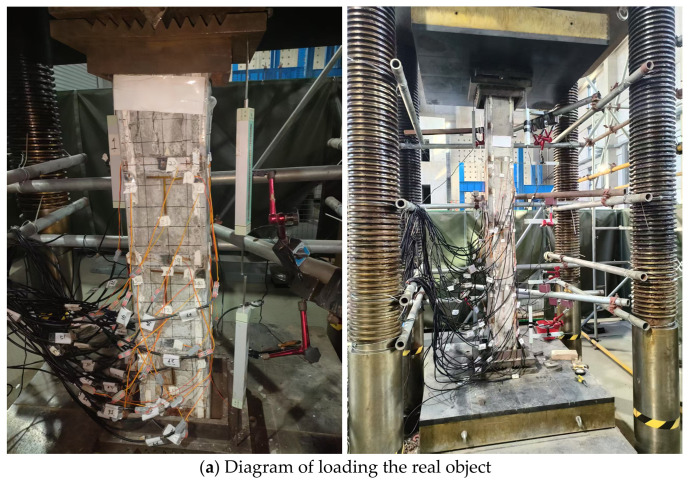

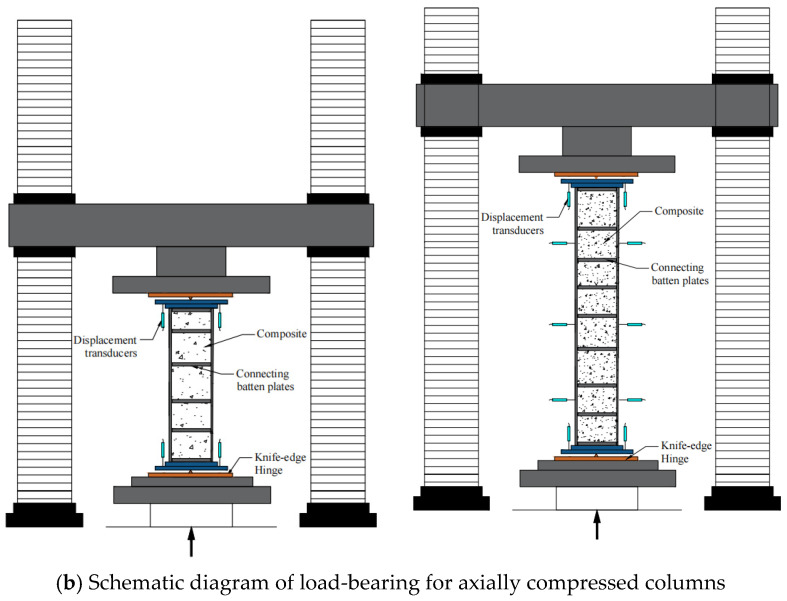
Test loading device.

The axial loading test was conducted using a 2000 T hydraulic servo press at the Structural Laboratory of Ningbo University of Technology. Both ends of the column specimen were designed as pin-supported boundaries, simulated using knife-edge hinge supports. The on-site setup is illustrated in Figure 6.

**Figure 7:**
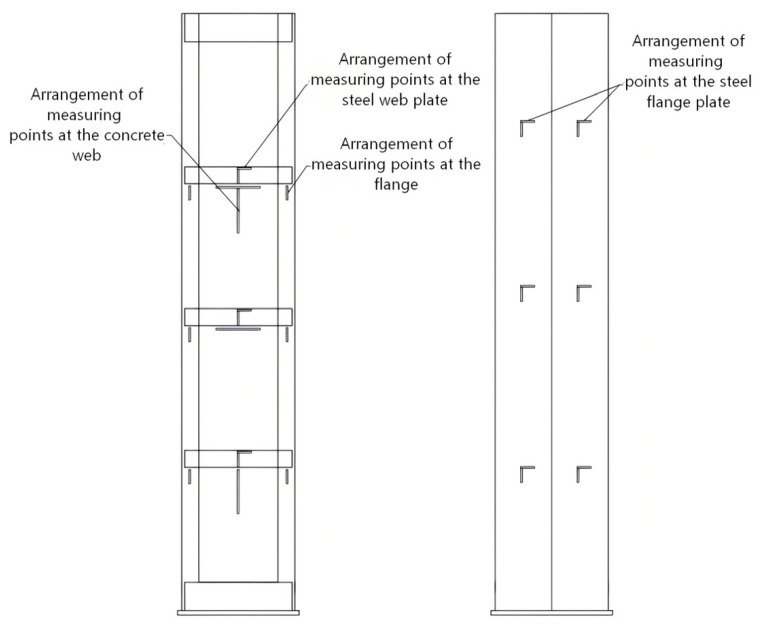
Detail drawing of reinforcement.

For the axially compressed short columns, only two vertical displacement transducers were arranged perpendicularly at the lower end of the specimen to determine axial deformation. For the long column specimens subjected to axial compression, in addition to installing 2 vertical displacement transducers each at the top and bottom to measure longitudinal deformation, 2 horizontal displacement transducers were also placed at the column top, at 1/4, 1/2 and 3/4 of the column height, as well as at the column base to monitor lateral displacement of the specimen at different heights. The specific arrangement is shown in Figure 7. Meanwhile, to obtain the stress distribution state across the specimen’s cross-section and to verify the alignment of the specimen, a number of electrical resistance strain gauges were systematically arranged at the quarter-height, mid-height and three-quarter-height sections. The layout scheme is also illustrated in Figure 7.

During the test preparation stage, the center of the lower hinged support was first determined as the reference point of the loading axis and the center of the upper knife-edge hinge was marked accordingly. The horizontal offset between the upper and lower center points was then measured and corresponding marks were made on the upper and lower end plates of the specimen to control the initial installation deviation. The specimen was subsequently lifted slowly into the testing machine and the centroid mark on the bottom end plate was aligned with the center of the lower support before being seated steadily. After positioning, the vertical alignment of the specimen was checked along the two mutually perpendicular directions corresponding to the major and minor axes, with particular attention paid to whether the specimen axis coincided with the reference point and whether the side edges remained parallel to the plumb line. If necessary, thin steel shims were inserted between the lower support and the specimen base to make fine adjustments. After alignment was completed, displacement transducers were installed and the strain-gauge wires were connected, followed by preloading. The preload was taken as 5% of the estimated ultimate load and was applied slowly and uniformly, with the load held for no less than 2 min, in order to eliminate contact gaps, check the working condition of the loading system, measuring instruments and data acquisition system and further verify the alignment condition and loading uniformity. Meanwhile, the specimen was inspected for any obvious inclination and the longitudinal strain readings on both sides were checked to confirm that the relative strain difference remained within the allowable tolerance. After all conditions were confirmed to be normal, the load was gradually released to zero.

**Figure 8:**
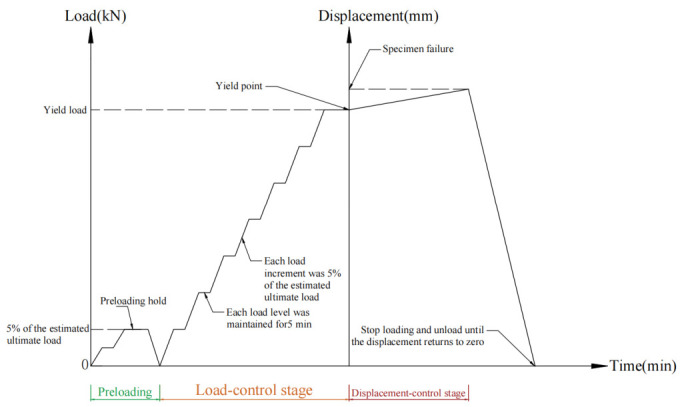
Loading protocol schematic diagram.

Formal loading was then conducted in two stages, namely load control and displacement control. Before yielding, the specimen was loaded in increments, with each load level taken as approximately 5% of the estimated ultimate load. As the specimen approached yielding, the load increment was appropriately reduced to facilitate accurate identification of the yield point. Each load level was maintained for no less than 2 min and the relevant data were recorded and crack development was observed after the deformation became stable. When a clear inflection point appeared in the load–displacement curve or when the longitudinal strain reached the steel yield strain, the specimen was considered to have entered the yielding stage. Thereafter, the loading mode was switched to displacement control and slow continuous loading was applied until specimen failure occurred or the load-carrying capacity decreased to the prescribed limit. The detailed loading procedure is illustrated in Figure 8.

## 3. Experimental Results and Analysis

A total of 12 axially loaded specimens were designed in this study, including 10 DCPEC columns and 2 bare steel built-up columns. The specimens were arranged to investigate several key influencing factors, including the connection method, steel wall thickness, member slenderness ratio and the number of connecting steel plates or welds and representative single-variable comparison groups were established to identify the effects of each parameter on the failure mode, load–displacement response and ultimate load-carrying capacity. Among these variables, the slenderness ratio was controlled by varying the member length and was taken as 17.32, 25.98, 34.64 and 41.57, respectively, so as to cover the stability variation range from stub columns to relatively slender columns. The connection-detail parameters were adjusted by changing the number of connecting plates or weld points, thereby altering the connection density between the two C-section steels along the column height and enabling evaluation of the influence of connection details on the overall composite action. The detailed grouping of the specimens is presented in Table 4.

### 3.1. Double C-Section Pure Steel Columns

#### 3.1.1. Test Procedure and Failure Modes

**Figure 9:**
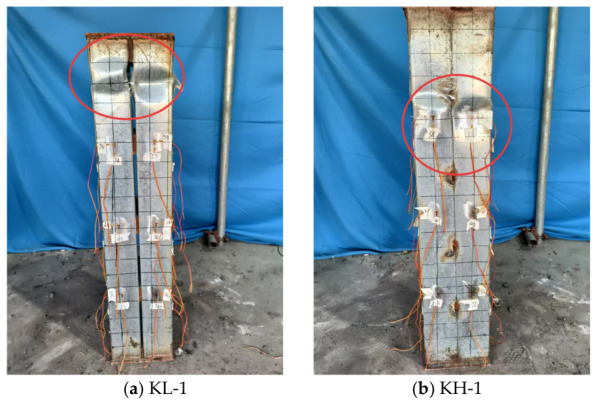
Typical failure mode of the axially compressed pure steel short columns.

The bare steel short-column specimens were designated as KL-1 and KH-1, in which KL-1 adopted bolted connections and KH-1 adopted welded connections. During the initial loading stage, no obvious abnormal response was observed in either specimen. When the applied load reached approximately 30% of the ultimate load, specimen KL-1 emitted a distinct audible sound. As the load further increased to about 70% of the ultimate load, local bulging deformation appeared in the web at a location approximately 200 mm from the top, indicating the onset of local buckling. The flange then gradually bulged outward and the specimen finally failed by distortional buckling. By contrast, specimen KH-1 also remained stable during the initial loading stage. When the load reached approximately 40% of the ultimate load, an audible sound was detected. As the load increased to about 70% of the ultimate load, local bulging occurred in the section near the top region, followed by flange out-of-plane deformation and torsional instability. The typical failure modes are shown in Figure 9.

#### 3.1.2. Load–Vertical Displacement Curve

**Figure 10:**
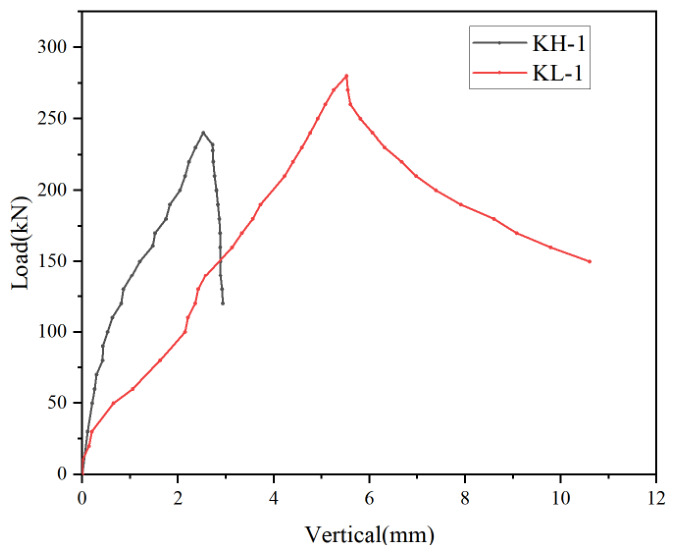
Load–Vertical displacement curve of pure steel columns.

In the comparative tests on pure steel short columns, the ultimate load of the bolted connection specimen was significantly higher than that of the welded connection specimen, indicating that, for double-limb built-up cold-formed thin-walled C-section steel columns, the bolted connection is more favorable for the full development of the overall mechanical performance of the member. As indicated by the load–displacement curves in Figure 10, the ultimate load of specimen KL-1 reached 282 kN, which was approximately 16% higher than that of specimen KH-1 at 243 kN. In terms of failure mode, KL-1 was mainly characterized by the development of distortional buckling following local bulging, whereas KH-1 exhibited pronounced twisting deformation after the onset of local buckling, indicating that the welded connection was more likely to induce coupled instability involving local buckling and torsion. The reason is that, with the aid of connecting steel plates, the bolted connection can provide a clearer and more stable load transfer path between the two C-section steel limbs, thereby enhancing the collaborative action of the cross-section and restraining relative deformation. By contrast, the welded connection in this study was mainly realized by discrete weld-point restraints, resulting in a non-uniform local stiffness distribution. In addition, since cold-formed thin-walled steel is sensitive to initial imperfections and residual stresses, local instability is more likely to occur prematurely under axial compression and subsequently develop into torsional instability. Overall, under the test conditions considered in this study, the bolted connection more effectively exploits the advantages of the overall stable load-carrying behavior of double-limb built-up thin-walled pure steel columns.

### 3.2. DCPEC Columns

#### 3.2.1. Test Observations of DCPEC Short Columns Under Axial Compression

**Figure 11:**
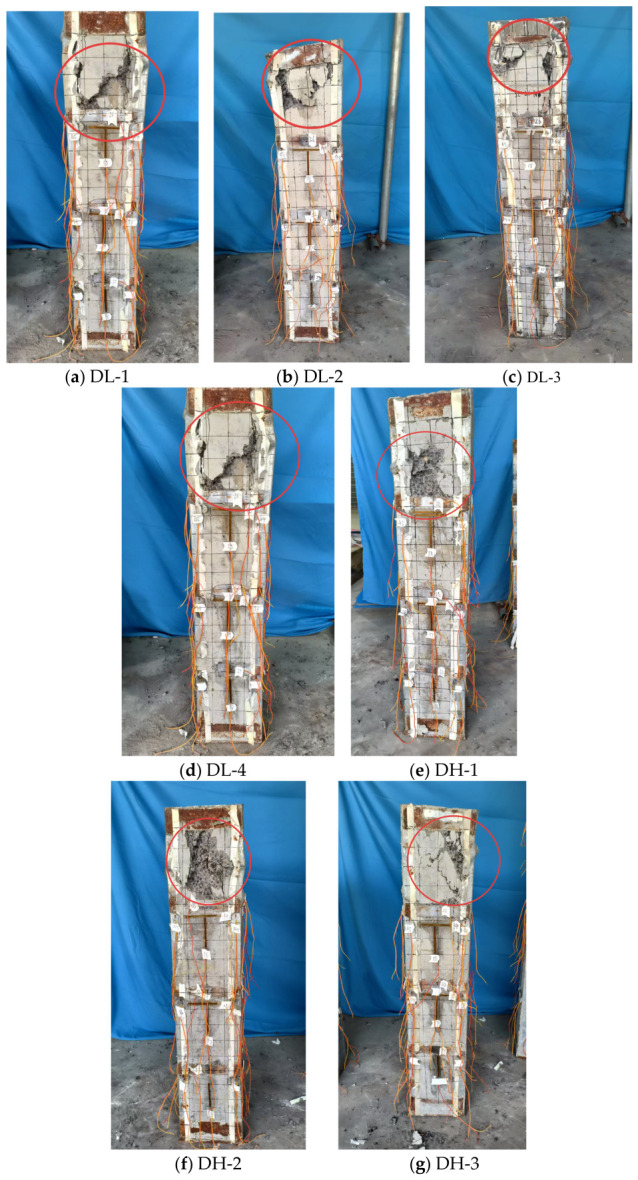
Typical failure mode of the axially compressed short columns.

Compared with the bare steel columns, the failure evolution of the seven DCPEC axially loaded stub column specimens during the loading process exhibited distinct stage characteristics. According to the test observations shown in Figure 11 and the corresponding load levels, the compressive failure process can be divided into the following three typical stages:

(1) Initial cracking stage (approximately 30–50% of the ultimate load):

When the load reached approximately 30–50% of the ultimate load, all specimens generally produced obvious sounds accompanied by the formation of the first visible cracks. This stage indicates the initiation and propagation of internal microcracks in the concrete, together with local damage to the steel–concrete interfacial bond. Specifically, DL-1 produced a distinct sound at 475 kN (approximately 40% of the ultimate load) and a fine crack appeared on the front face at 15 cm from the top; DL-3 developed a crack on the front face at approximately 10 cm from the bottom at 329 kN (approximately 30% of the ultimate load); DH-1 developed a crack on the front face at approximately 10 cm from the top at 317 kN (approximately 30% of the ultimate load); and DH-2 produced a slight sound at 295 kN (approximately 30% of the ultimate load), followed by a crack on the front face at approximately 5 cm from the bottom. It can thus be seen that the initial damage of the stub columns mainly occurred in the end regions of the columns, within approximately 10 cm from the top or bottom, which is mainly attributed to the difference between the end restraint conditions and those along the column body, as well as the tendency for stress concentration to occur at the ends during the early stage of loading. The load levels corresponding to the initial damage stage in the welded specimens DH-1, DH-2 and DH-3 (approximately 30% of the ultimate load) were slightly lower than that of the bolted specimen DL-1 (approximately 50% of the ultimate load), indicating that the welded configuration may lead to faster stress redistribution or more pronounced local stress concentration during the early stage of loading.

(2) Steel buckling and crack propagation stage (approximately 50–80% of the ultimate load):

When the load increased to approximately 50–80% of the ultimate load, obvious local buckling of the steel sections occurred in all specimens, mainly manifested as local outward bulging or inward denting. Meanwhile, the concrete cracks expanded significantly and spalling began to occur. This stage represents the critical stage in the failure evolution of the stub columns, during which the confining effect of the steel sections on the concrete gradually weakened, the stress within the cross-section was redistributed and the composite action between steel and concrete was correspondingly reduced. Specifically, at 935 kN (approximately 90% of the ultimate load), DL-1 exhibited outward bulging of the steel section at 10 cm from the bottom on the left side, accompanied by concrete cracking and spalling. The relatively high corresponding load level may be related to its stronger overall configuration with five connecting steel plates. DL-2 exhibited outward bulging of the steel section at 10 cm from the top on the side face at 854 kN (approximately 70% of the ultimate load), while multiple cracks appeared and the existing cracks further propagated. DL-3 exhibited inward denting of the steel section at 10 cm from the bottom on the side face at 726 kN (approximately 60% of the ultimate load) and the cracks continued to propagate. Among the remaining specimens, DL-4 and DH-1 exhibited outward bulging of the steel section on the side face at 549 kN and 534 kN, respectively, accompanied by aggravated cracking, corresponding to approximately 80% and 50% of the ultimate load, respectively. For DH-2, when the load reached 534 kN, i.e., approximately 60% of the ultimate load, outward bulging of the steel section occurred at 15 cm from the bottom on the side face, accompanied by concrete cracking. For DH-3, a crack first appeared at 5 cm from the bottom on the rear face at 670 kN, i.e., approximately 60% of the ultimate load; when the load increased to 980 kN (approximately 80% of the ultimate load), multiple bulging deformations appeared in the steel section and large-area concrete spalling occurred. The test observations indicate a strong correlation between the steel buckling locations and the initial crack locations, as the buckling of most specimens occurred near the regions where the initial cracks first appeared, suggesting that the locations of early damage were often the critical regions governing subsequent failure evolution. DL-4, with a smaller number of connecting steel plates (three bolted connecting plates) and DH-2, with a smaller number of welds (three welded points), exhibited more pronounced local deformation during the buckling development stage, reflecting the important influence of the connection configuration on the lateral restraint capacity and local stability of the thin-walled steel sections.

(3) Peak load and failure stage (upon reaching the ultimate load):

After reaching the peak load, the load-carrying capacity of all specimens decreased rapidly and the failure was generally characterized by complete crushing of the concrete at the bottom or end regions, accompanied by further aggravation of steel buckling. Specifically, when DL-1 reached its peak load of 1028 kN, the concrete at the bottom of the front face was crushed and the buckling of the steel section became significantly more severe. DL-2 and DL-3 failed when the ultimate loads reached 1098 kN and 1240 kN, respectively, with crushing of the concrete at the bottom accompanied by steel buckling. When DH-3 reached the ultimate load of 1110 kN, the concrete at the bottom of the front face was crushed and the overall inclination of the column body occurred. From the ultimate load results of the specimens, it can be seen that DL-2 (2.5 mm) and DL-3 (3.0 mm), which had larger wall thicknesses, exhibited higher load-carrying capacities than DL-1 (2.0 mm), indicating that the wall thickness of the steel section made a significant contribution to the load-carrying capacity of the member. The load-carrying capacity of DL-4, which had the smallest number of connecting steel plates (three plates), was significantly lower than that of DL-1, which had the same wall thickness but five connecting plates, indicating that the number of connecting steel plates had an important influence on the restraining effect and overall integrity of the member. Among the welded specimens DH-1, DH-2 and DH-3, DH-3, which had the largest number of welds (11 welded points), exhibited the highest load-carrying capacity, but overall column inclination occurred at failure, which may be related to the heat-affected effect of welding or welding residual stresses. At failure, the concrete crushing locations were mainly concentrated at the bottom ends of the columns and were highly consistent with the crack initiation locations and steel buckling locations, further indicating that the failure of the stub columns exhibited pronounced localization and end effects.

#### 3.2.2. Test Observations of DCPEC Long Columns Under Axial Compression

**Figure 12:**
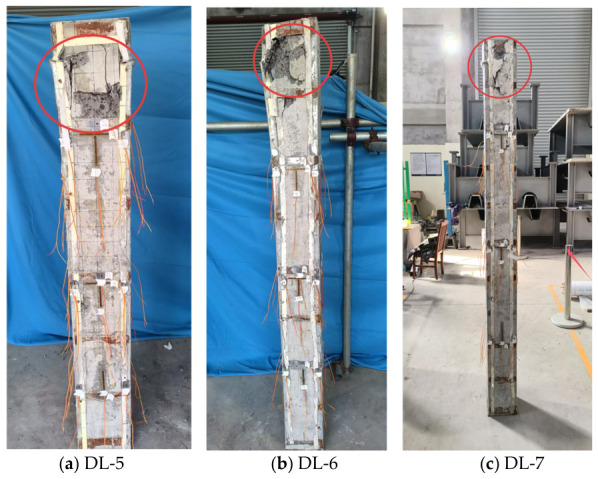
Typical failure mode of the axially compressed long columns.

The failure process of the axially loaded slender columns was more rapid and exhibited greater abruptness than that of the stub columns. According to the test observations shown in Figure 12, their stage characteristics can be summarized as follows:

(1) Initial cracking stage (approximately 30–70% of the ultimate load):

During the early loading stage, the slender columns also exhibited audible sounds and minor cracks; however, the load level at which they appeared was significantly affected by the slenderness ratio, resulting in a relatively wide variation range. DL-5 (slenderness ratio of 25.98) produced a slight sound at 400 kN (approximately 30% of the ultimate load), indicating that damage occurred relatively early but developed slowly. DL-6 (slenderness ratio of 34.64) emitted a relatively loud sound at 900 kN (approximately 80% of the ultimate load), indicating that damage appeared relatively late but then expanded rapidly. DL-7 (slenderness ratio of 41.57) exhibited audible sounds and fine cracks at 690 kN (approximately 70% of the ultimate load) and entered the failure stage shortly after the occurrence of damage. These observations indicate that, with increasing slenderness ratio, the slender columns remained relatively stable during the early loading stage and exhibited less obvious test phenomena, with the onset of damage being relatively delayed; however, once damage occurred, the failure process accelerated markedly, which is closely related to the mechanisms characteristics of stability-controlled members. For DL-7, which had the largest slenderness ratio, the first audible sound did not appear until the load reached approximately 75% of the ultimate load and almost no obvious precursor was observed before that, reflecting the pronounced abruptness of failure in slender columns.

(2) Steel buckling and crack propagation stage (approximately 70–95% of the ultimate load):

When the load reached 70–95% of the ultimate load, damage in the slender columns developed significantly faster and the lateral displacement began to increase markedly. For DL-5, a small amount of concrete spalling appeared on the member surface at 840 kN (approximately 70% of the ultimate load), indicating the onset of visible damage; when the load increased to 970 kN (approximately 80% of the ultimate load), extensive concrete spalling occurred on the column surface, accompanied by slight bulging of the steel section and damage development accelerated. For DL-6, after a relatively loud sound was emitted at 900 kN (approximately 80% of the ultimate load), bulging of the steel section occurred at 10 cm from the top on the left side at 940 kN (approximately 90% of the ultimate load), while a large number of cracks rapidly propagated on both sides. The load increment from the occurrence of the sound to steel buckling was only 40 kN, indicating extremely rapid damage evolution. When the load reached 1030 kN (approximately 95% of the ultimate load), extensive concrete spalling occurred and cracks continued to increase. For DL-7, after cracks appeared at 690 kN (approximately 70% of the ultimate load), a small amount of concrete spalling appeared on the member surface at 900 kN (approximately 95% of the ultimate load) and bulging of the steel section occurred at 15 cm from the top on the left side. The load increment from crack initiation to steel buckling was 210 kN, but the deformation increased sharply when the load approached the ultimate load. During this stage, the lateral deflection of the slender columns increased significantly, leading to an increase in the sectional bending moment and a further concentration of compressive stress in the compression zone, thereby forming a positive-feedback instability development process.

(3) Peak load and failure stage (upon reaching the ultimate load):

After the slender columns reached the peak load, the load-carrying capacity decreased rapidly and the failure process was relatively short. DL-5 had an ultimate load of 1200 kN and failure was characterized by concrete crushing at the top of the front face and bulging of the steel section at 10 cm from the top. DL-6 had an ultimate load of 1080 kN and failure was characterized by concrete crushing accompanied by dense crack propagation. DL-7 had an ultimate load of 918 kN and failure was characterized by concrete crushing at the top of the front face. As the slenderness ratio increased, the ultimate load-carrying capacity of the specimens showed a clear decreasing trend: the ultimate load of DL-5 (slenderness ratio of 25.98) was 1200 kN, that of DL-6 (slenderness ratio of 34.64) was 1080 kN, representing a decrease of approximately 10% and that of DL-7 (slenderness ratio of 41.57) was 918 kN, representing a decrease of approximately 24% compared with DL-5, which fully demonstrates that the slenderness ratio has a significant controlling effect on the stability-related load-carrying capacity of the member. At failure, all specimens exhibited the combined action of concrete crushing in the compression zone and steel buckling and the buckling locations were mostly located at a certain distance from the column ends (approximately 10–15 cm from the top), rather than at the column ends themselves. This is in clear contrast to the failure characteristics of stub columns, where damage was mainly concentrated at the ends and reflects the failure characteristics of overall flexural instability. The failure locations of both DL-6 and DL-7 were located at a certain distance from the top, which is generally consistent with the mechanisms’ behavior of pin-ended compression members, where relatively large bending moments develop along the column height; however, owing to the influence of the slenderness ratio level and local initial imperfections, a certain deviation occurred in the actual failure location.

#### 3.2.3. Load–Vertical Displacement Curve

**Figure 13:**
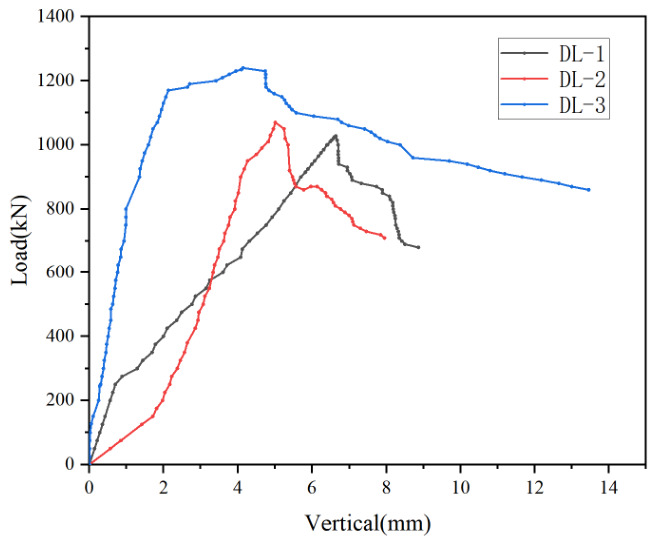
Comparison of load–vertical displacement curves under different wall thicknesses.

**Figure 14:**
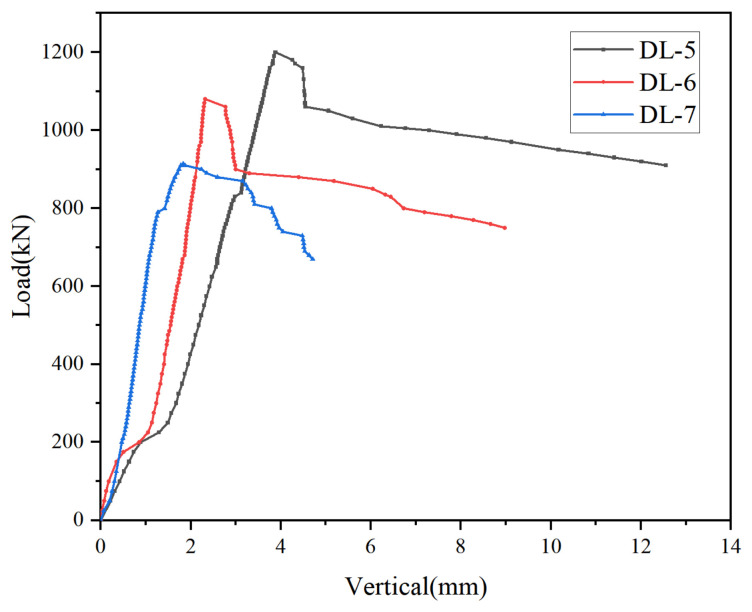
Comparison of load–vertical displacement curves under different slenderness ratios.

**Figure 15:**
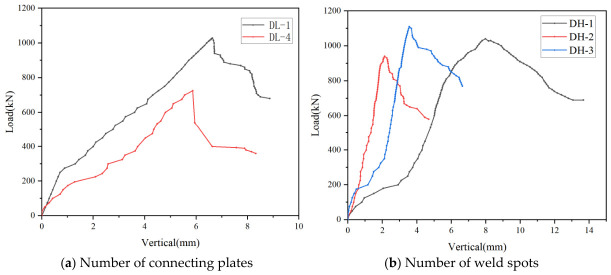
Comparison of load–vertical displacement curves under different numbers of connecting plates and weld spots.

(1) Wall thickness: To investigate the influence of the wall thickness of the C-section steel on the load-carrying capacity of DCPEC members, three specimens with wall thicknesses of 2.00 mm (DL-1), 2.50 mm (DL-2) and 3.00 mm (DL-3) were selected for comparative analysis. As indicated by the load–vertical displacement curves shown in Figure 13, the ultimate load-carrying capacity of the specimens increased significantly with increasing wall thickness of the steel section. Specifically, the ultimate load of specimen DL-2 was 1098 kN and that of specimen DL-3 was 1240 kN, representing increases of approximately 5% and 21%, respectively, compared with specimen DL-1, whose ultimate load was 1028 kN. These results indicate that a greater wall thickness of the steel section leads to a higher ultimate load-carrying capacity of the member.

(2) Specimen slenderness ratio: The slenderness ratio of the member had a significant influence on its load-carrying capacity. Specimens with slenderness ratios of 25.98 (DL-5), 34.64 (DL-6) and 41.57 (DL-7) were selected for comparison. The corresponding specimen lengths were 1500 mm for DL-5, 2000 mm for DL-6 and 2400 mm for DL-7. As shown in Figure 14, under axial compression, the ultimate load of the members decreased with increasing slenderness ratio. Specifically, when the slenderness ratio increased from 25.98 (DL-5) to 34.64 (DL-6), the ultimate load decreased from 1200 kN to 1080 kN, corresponding to a reduction of 11%. When the slenderness ratio further increased to 41.57 (DL-7), the ultimate load dropped to 918 kN, which was 30.6% lower than that of DL-5. These results indicate that an increase in slenderness ratio leads to a reduction in the ultimate load of the member and has a detrimental effect on its mechanical performance.

(3) Number of connecting plates and weld spots: The number of connecting plates in the C-section steel member had a significant influence on its load-carrying capacity. Under otherwise identical conditions, specimens DL-1 (with 5 connecting plates) and DL-4 (with 3 connecting plates) were selected for comparison. As shown by the load–vertical displacement curves in Figure 15a, the ultimate load of DL-1 was approximately 40% higher than that of DL-4, indicating that increasing the number of connecting plates can effectively enhance the load-carrying capacity of the member. The number of weld spots was also an important parameter affecting the load-carrying capacity. With all other conditions kept identical, specimens DH-1 (with 6 weld spots), DH-2 (with 3 weld spots) and DH-3 (with 11 weld spots) were selected for comparison. According to the load–vertical displacement curves shown in Figure 15b, specimen DH-3 exhibited the highest ultimate load, which was approximately 7% and 18% higher than those of DH-1 and DH-2, respectively. These results indicate that, under the condition of maintaining the same weld length, an appropriate increase in the number of weld spots can further improve the load-carrying capacity of the member.

### 3.3. Mechanism Analysis

(1) Effect of connection type on axial compressive behavior.

The superiority of the bolted connection over the welded connection does not primarily arise from a simple difference in connection strength, but rather from the fact that the former is more favorable for establishing a stable and integrated collaborative load-resisting system between the two cold-formed thin-walled C-section steel limbs. The above experimental results indicate that the bolted specimens exhibited higher load-carrying capacity and a relatively milder instability evolution process. By contrast, once local buckling occurred in the welded specimens, it was more likely to further develop into coupled torsional instability. This phenomenon indicates that the connecting steel plate–bolt system can provide a clearer, more uniform and more stable load transfer path between the two limbs, thereby more effectively restraining relative opening, relative slip and twisting. In comparison, the restraint provided by discrete weld-point connections is relatively non-uniform and therefore more likely to induce local deformation concentration and instability propagation.

(2) Delaying effect of lightweight aggregate concrete on local and distortional buckling.

The experimental observations show that local bulging of the steel web in the pure steel specimen KL-1 occurred at approximately 70% of the ultimate load, followed by outward bulging of the flange and the development of distortional buckling. After the peak load was reached, the load dropped rapidly without an obvious plateau stage. In contrast, the composite short column DL-1 exhibited only a slight cracking sound at approximately 30% of the ultimate load, while steel plate bulging did not occur until about 90% of the ultimate load, accompanied by concrete cracking and spalling. The descending branch was reached only after the load attained 1028 kN and except for the locally crushed zone near the top, the concrete in the remaining regions remained basically intact. This comparison indicates that, after concrete infill, the inner side of the steel plate changed from an unrestrained cavity condition to a continuously supported condition and the out-of-plane deformation of the web and flanges was effectively restrained. As a result, the restraint that must be overcome for local outward bulging was significantly increased and both local buckling and the subsequent distortional buckling were delayed. From the parametric results, local stability of the steel plate remained the key factor governing the load-carrying capacity, although the concrete could significantly weaken its adverse effect. The wall-thickness comparison results of the specimens presented in this paper show that the ultimate loads of DL-1, DL-2 and DL-3 were 1028, 1098 and 1240 kN, respectively. When the wall thickness of the C-section steel increased from 2.0 mm to 2.5 mm and 3.0 mm, the load-carrying capacity increased by approximately 8.2% and 9.9%, respectively, corresponding to overall increases of about 10% and 16% relative to DL-1. This indicates that, even under the condition of concrete infill, the local stability of the steel plate still determines the upper limit of the member load-carrying capacity. At the same time, it also demonstrates that the concrete indeed delayed the development of local instability, because without internal support, cold-formed thin-walled steel plates would exhibit greater sensitivity to variations in wall thickness.

(3) Interaction mechanism between steel restraint and lightweight aggregate concrete.

The interaction between steel restraint and lightweight aggregate concrete is essentially a bidirectional collaborative working mechanism, in which “the steel restrains the concrete, while the concrete supports the steel plate”. During compression, the steel plate provides confinement to the lateral deformation of the concrete, thereby enhancing the contribution of the concrete to the load-carrying capacity of the member. In turn, through interfacial contact and friction, the concrete provides reverse support to the bulging deformation of the web and flanges, thereby delaying local instability of the steel plate and improving the overall stiffness of the cross-section.

(4) Effect of weld-spot quantity on load-carrying capacity.

When the number of weld points increases to a certain extent, its beneficial effect tends to become limited, mainly because the governing factor gradually shifts from “insufficient connection” to “instability control of the member itself”. The ultimate loads of DH-2, DH-1 and DH-3 were 942, 1040 and 1110 kN, respectively. When the number of weld points increased from 3 to 6, the load-carrying capacity increased by approximately 10.4%; however, when it was further increased from 6 to 11 or to continuous welding, the increase was only about 6.7%. These results indicate that, when the number of weld points is small, increasing the weld density can significantly improve the collaborative load-resisting performance of the two limbs. However, once the connection is sufficient to ensure overall force transfer, further increasing the number of weld points can no longer fundamentally alter the dominant failure modes, such as local plate buckling, crushing of the end concrete and overall weak-axis flexural buckling. Consequently, the marginal gain is markedly reduced.

(5) Influence of local steel plate bulging on concrete crushing.

The experimental observations indicate that local steel plate bulging is not merely an accompanying phenomenon unrelated to concrete crushing, but rather an important transitional stage between the two. In specimen DL-1, steel plate bulging first occurred at approximately 90% of the ultimate load, accompanied by concrete cracking and continuous spalling and the top concrete was crushed after the peak load was reached. In specimen DL-5, surface spalling first occurred at approximately 70% of the ultimate load, followed by slight steel plate bulging at about 80% and crushing of the top concrete after the peak load of 1200 kN was reached. In specimen DL-6, steel plate bulging occurred at approximately 85% of the ultimate load together with multiple cracks, followed by failure. The underlying mechanism is that once local bulging occurs in the steel plate, the lateral confinement provided by this region to the concrete is significantly weakened, while the lateral deformation of the concrete and local stress concentration are correspondingly intensified. Meanwhile, local steel plate bulging also alters the internal force distribution within the cross-section and induces additional eccentricity and local second-order bending moments, thereby accelerating crack propagation and surface spalling and ultimately triggering local crushing of the concrete. It can therefore be concluded that local steel plate bulging is not a subsidiary phenomenon of concrete crushing, but rather an important mechanism stage that promotes deterioration of interfacial collaborative action, aggravates stress concentration and ultimately triggers local concrete crushing.

## 4. Finite Element Simulation Results and Analysis

### 4.1. Determination of Constitutive Relations

At present, extensive experimental studies and theoretical analyses on lightweight aggregate concrete have been carried out by scholars both in China and abroad and systematic achievements have been made in such aspects as constitutive relationships, mechanical performance indices and design calculation methods, some of which have been incorporated into current design codes. In view of the differences in parameter values and applicable conditions of the constitutive models for lightweight aggregate concrete adopted in different studies and codes, this paper, through a comparative analysis of the theoretical basis and applicability of various constitutive models, adopts the uniaxial compressive constitutive relationship for lightweight aggregate concrete specified in JGJ/T 12-2019, Technical Standard for Application of Lightweight Aggregate Concrete Structures [21] and its typical stress–strain curve is expressed by Equation (1):

(1) $$\begin{array}{l} When\;\varepsilon \leq \varepsilon_{0}:\;\sigma_{c}=f_{c}\left[1.5\left|\frac{\varepsilon_{c}}{\varepsilon_{0}}\right|-0.5{\left|\frac{\varepsilon_{c}}{\varepsilon_{0}}\right|}^{2}\right]\\ When\;\varepsilon <\varepsilon_{0}<\varepsilon_{cu}:\sigma_{c}=f_{c} \end{array}$$

where $\sigma_{c}$ is the compressive stress of lightweight aggregate concrete at a compressive strain of $\varepsilon_{c}$; $f_{c}$ is the design value of the axial compressive strength of lightweight aggregate concrete; $\varepsilon_{0}$ is the compressive strain of lightweight aggregate concrete when the compressive stress just reaches $f_{c}$; $\varepsilon_{cu}$ is the ultimate compressive strain of lightweight aggregate concrete in the normal section. Under non-uniform compression, $\varepsilon_{cu}$ is taken as 0.0033; under axial compression, it is taken as $\varepsilon_{0}$.

**Figure 16:**
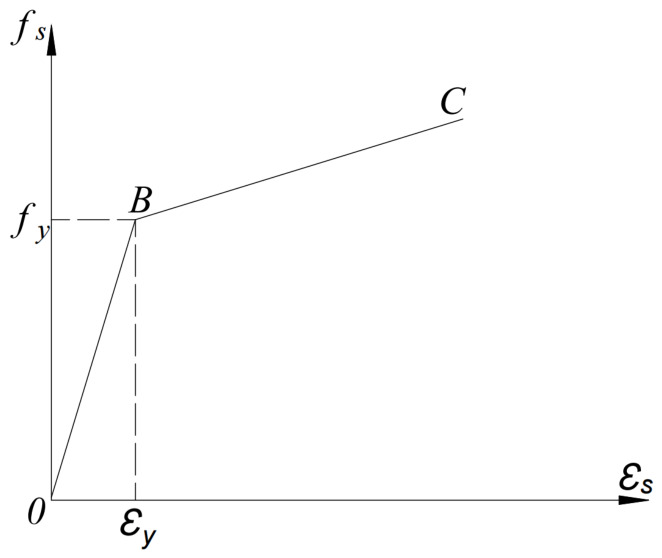
Constitutive relationship curve of steel.

According to the experimental results, the yield strength, elastic modulus and Poisson’s ratio of the steel were determined as 207 MPa, 210.6 GPa and 0.3, respectively. The constitutive model for the steel section was selected from Refs. [22,23] and its mathematical expression is given in Equation (2). The corresponding constitutive relationship curve is shown in Figure 16, while Equation (2) represents its mathematical formulation.

(2) $$\begin{array}{l} When\;\varepsilon >\varepsilon_{1}:\;\sigma_{s}=f_{y}+\frac{E_{s}}{150\left(\varepsilon -\varepsilon_{1}\right)}\\ When\;0<\varepsilon <\varepsilon_{1}:\;\sigma_{s}=E_{s}\varepsilon \end{array}$$

where $\varepsilon_{s}$ is the strain of the steel section; $\varepsilon_{y}$ is the yield strain of the steel section; $\varepsilon_{s,h}$ is the strain at the onset of strain hardening of the steel section; $f_{y}$ is the yield strength of the steel section; $\sigma_{s}$ is the stress of the steel section; $E_{s}$ is the elastic modulus of the steel section.

### 4.2. Model Establishment, Mesh Generation and Boundary Conditions

**Figure 17:**
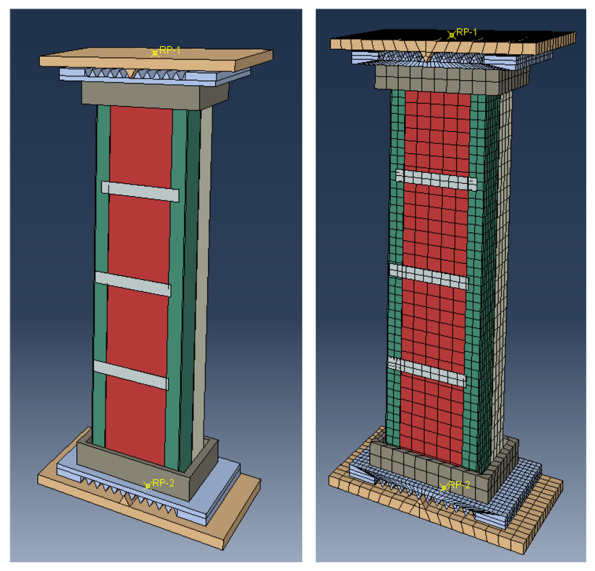
Establishment of finite element model.

The model was established based on the component design dimensions described above. The bottom knife-edge hinge support and end plates were modeled separately and subsequently assembled, as illustrated in Figure 17.

The cold-formed thin-walled C-section steel, connecting steel plates and end plates were modeled using four-node reduced-integration shell elements (S4R). These elements can accurately capture the coupled in-plane and out-of-plane deformation of thin-walled plate components under axial compression, as well as the local buckling and distortional buckling behavior, while maintaining relatively high computational efficiency without compromising accuracy. The lightweight aggregate concrete was modeled using eight-node reduced-integration solid elements (C3D8R) to reflect its three-dimensional compressive deformation and the contact interaction with the steel plates. The knife-edge hinge supports were modeled as analytical rigid bodies and coupling relationships were established between the supports and the end plates through reference points, so as to reduce the interference of local support deformation with the analysis of the primary structural response. The geometric dimensions of the model were kept consistent with those of the specimens.

The steel–concrete interface was simulated using surface-to-surface contact. The normal behavior was defined as “hard contact”, while the tangential behavior was modeled using a penalty friction formulation with a friction coefficient of 0.3, in order to account for interfacial contact force transfer and the restraint effect on relative slip. “Tie” constraints were adopted between the end plates at both ends and the steel members, as well as the concrete ends, to ensure coordinated force transfer at the member ends during loading. The welded connection regions were equivalently simulated using “Tie” constraints to represent the restraining effect of the welds on the collaborative action between the two C-section steel limbs. The boundary conditions were defined according to the test setup, in which both ends of the specimen were modeled as knife-edge hinged supports. Reference points were introduced at the centers of the upper and lower supports to apply boundary constraints and axial loading. The bottom reference point was restrained in translation to eliminate rigid-body motion, whereas the top reference point was restrained in the lateral translational directions while remaining free in the axial direction for loading. The rotational freedom at the specimen ends was not directly prescribed through the reference points but was realized through the contact interaction between the end plates and the knife-edge hinge supports. In this way, the actual pin-ended boundary condition of the test specimens could be represented more reasonably.

Considering that cold-formed thin-walled members are sensitive to initial geometric imperfections, the first-order buckling mode obtained from eigenvalue buckling analysis was extracted as the initial imperfection distribution and then introduced into the nonlinear analysis model, in order to more reasonably trigger local buckling and overall instability. The amplitude of the initial imperfection was taken as L/1000, where L is the member length. This treatment can more realistically reflect the local stability problems induced by initial imperfections during the actual compression process of cold-formed thin-walled members, without excessively amplifying the effect of initial deviations.

To determine an appropriate mesh size while balancing computational accuracy and efficiency, the short-column specimen DL-1 and the relatively longer specimen DL-6 were selected as representative members for mesh sensitivity analysis. Four mesh sizes, namely 30 mm, 25 mm, 20 mm and 10 mm, were adopted for the main body of the members, while the mesh sizes of the supports and end plates were correspondingly taken as 30 mm, 25 mm, 15 mm and 10 mm. The comparison indices included the ultimate load, the initial stiffness of the load–displacement curves, the peak displacement and the local buckling waveform. The results showed that the calculated results were significantly improved when the mesh size was refined from 30 mm to 25 mm. When the mesh was further refined to 20 mm, the variations in the ultimate load and initial stiffness became markedly smaller and the location of local steel plate bulging, as well as the dominant failure mode, remained essentially unchanged. Further refinement to 10 mm led to only limited improvement in the results, while the computational time increased substantially. Considering both computational accuracy and analysis efficiency, the final mesh size was determined as 20 mm for the main body of the members and 25 mm for the supports and end plates. These results indicate that the adopted mesh discretization is capable of stably capturing the load-carrying capacity and instability characteristics of DCPEC columns under axial compression.

### 4.3. Model Validation

**Figure 18:**
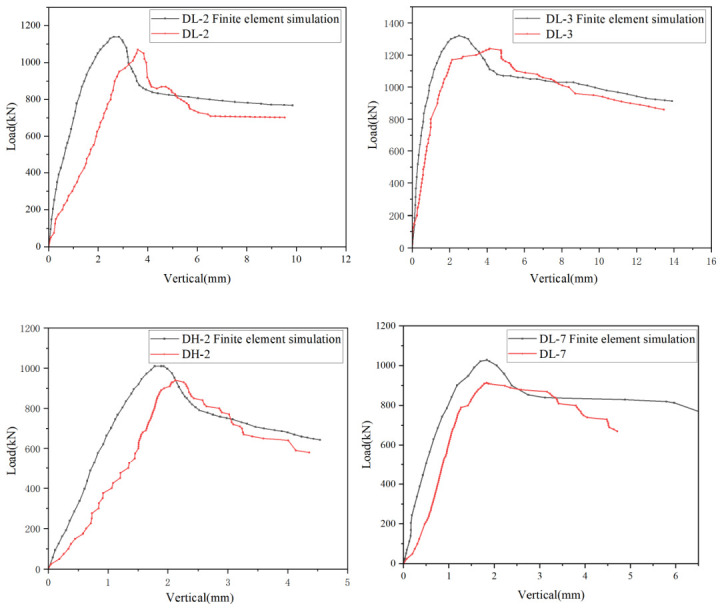
Comparison of load–vertical displacement curves.

**Figure 19:**
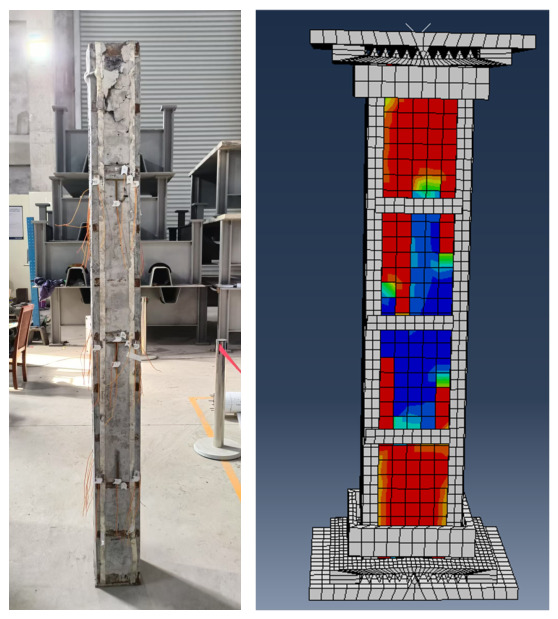
Comparison of failure modes between experimental and finite element analysis.

Before conducting the parametric analysis, in order to verify the reliability of the established finite element model, axial compression finite element simulations and axial compression tests were carried out for all specimens and the corresponding results were systematically compared. The model validation was mainly performed from three aspects, namely the load–vertical displacement response, failure mode and ultimate load-carrying capacity. First, by comparing the finite element curves with the experimental curves, the predictive capability of the model with respect to the elastic-stage stiffness, peak load and post-peak softening characteristics was evaluated and the comparison results for several representative specimens are shown in Figure 18. Second, the locations of local steel plate bulging, the outward bulging characteristics of the flanges and the crushing pattern of the end concrete predicted by the finite element model were compared with the experimental observations and the corresponding failure modes are presented in Figure 19. Finally, taking the relative error in ultimate load-carrying capacity as the quantitative evaluation index, the comparison between the finite element results and the experimental values is listed in Table 5. Overall, the finite element model reproduced the axial load–displacement response and the dominant failure characteristics of the specimens with reasonable accuracy. The relative error in ultimate load was within approximately 12.51% for all specimens, indicating that the established model is suitable for subsequent parametric analysis.

### 4.4. Finite Element Parametric Analysis

#### 4.4.1. Influence of Wall Thickness on Bearing Capacity

**Figure 20:**
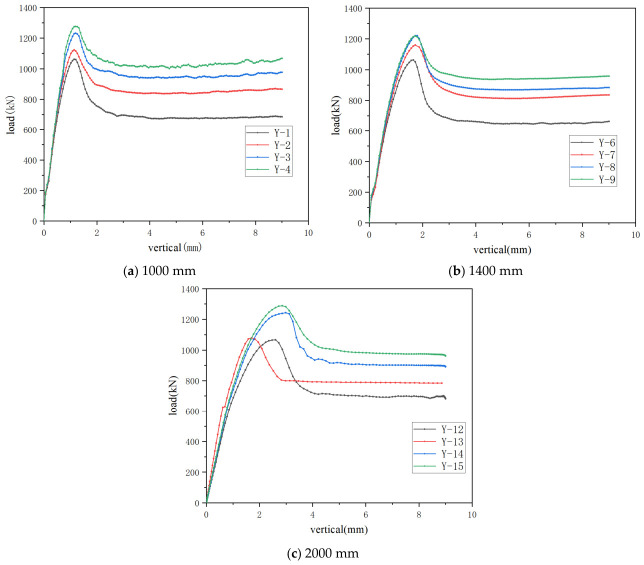
Comparison of load–vertical displacement curves for different wall thicknesses.

For the specimens with a height of 1000 mm, it can be seen from the results in Table 6 and Figure 20 that when the wall thickness increased from 1.50 mm to 2.00 mm, the ultimate load increased by 6.1%. When the wall thickness of the steel section increased from 1.50 mm to 3.00 mm, the ultimate load increased from 1051 kN to 1281 kN, corresponding to an increase of 21.9%. By contrast, when the wall thickness increased from 2.50 mm to 3.00 mm, the increase was 4.7%, indicating that the improvement in load-carrying capacity was more pronounced within the smaller wall-thickness range.

For the specimens with a height of 1400 mm, the ultimate load generally increased with the increase in wall thickness of the steel section, whereas the rate of increase gradually slowed down. When the wall thickness increased from 1.50 mm to 2.00 mm, the ultimate load increased from 1053 kN to 1164 kN, representing an increase of 10.5%. When the wall thickness was further increased to 2.50 mm and 3.00 mm, the increases were 5.0% and 1.8%, respectively. It can therefore be seen that, once the wall thickness exceeded 2.50 mm, the beneficial effect of further increasing the thickness on the load-carrying capacity gradually diminished.

For the specimens with a height of 2000 mm, the wall thickness of the steel section had a relatively significant influence on the ultimate load-carrying capacity. When the wall thickness increased from 1.50 mm to 2.00 mm, the ultimate load increased by only 6.3%, indicating a limited improvement. However, when the wall thickness increased from 2.00 mm to 2.50 mm, the ultimate load increased sharply from 1079 kN to 1243 kN, corresponding to an increase of 15.2%. When the wall thickness was further increased to 3.00 mm, the increase was 3.7%. These results indicate that, for this specimen height, there existed a distinct wall-thickness threshold of 2.50 mm, beyond which the improvement in load-carrying capacity tended to become more gradual.

#### 4.4.2. Influence of Slenderness Ratio on Bearing Capacity

**Figure 21:**
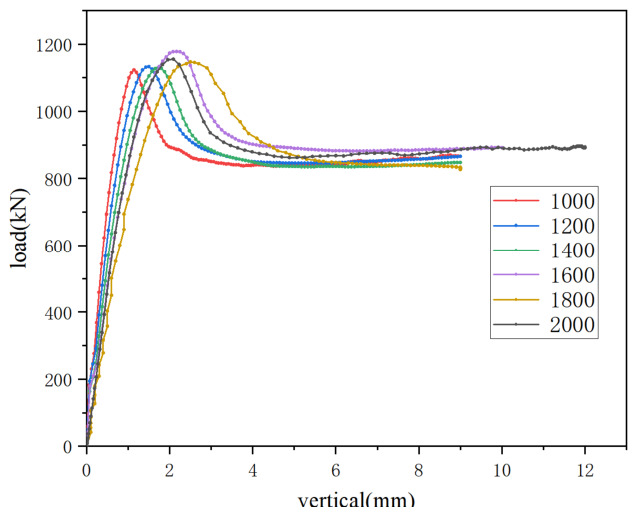
Comparison of load–vertical displacement curves.

**Figure 22:**
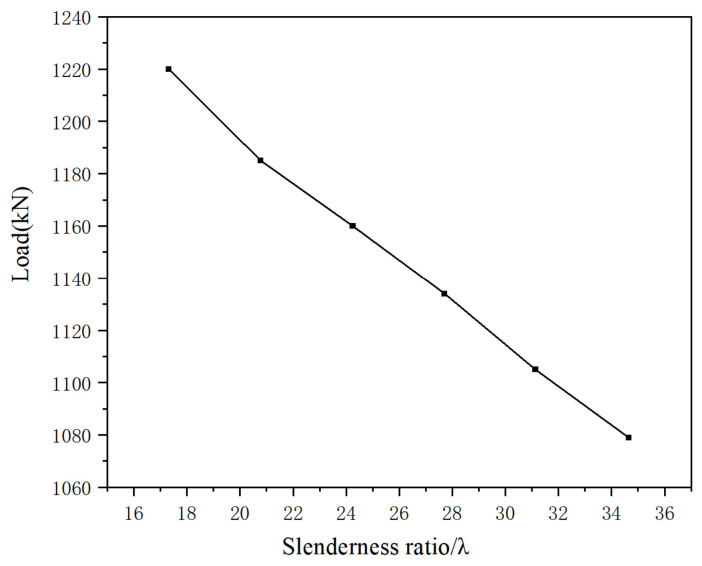
Load-slenderness ratio curve under different slenderness ratios.

The slenderness ratio of the member significantly affects its axial compression bearing capacity. Under the condition of keeping all other parameters constant, the slenderness ratio was varied within the range of 17.32 to 34.64 by altering the member length. The ultimate bearing capacities corresponding to each slenderness ratio are listed in Table 7. As shown in Figure 21 and Figure 22, under axial compression, the ultimate load of members with different cross-sectional forms decreases as the slenderness ratio increases. Taking simulated specimens Y-2 and Y-13 as an example, when the slenderness ratio increases from 17.32 to 34.64, their ultimate load decreases from 1220 kN to 1079 kN, a reduction of approximately 13%. This demonstrates that the slenderness ratio has a significant influence on the ultimate load of such members; the higher the slenderness ratio, the lower the bearing capacity of the member.

## 5. Conclusions

Based on the axial compression tests and the corresponding Abaqus finite element analysis, this study systematically investigated the failure modes, load–displacement responses and key parameter effects of double C-section partially encased composite columns (DCPEC columns). The results show that this type of member can effectively exploit the collaborative action between cold-formed thin-walled steel and lightweight aggregate concrete, thereby achieving relatively high axial load-carrying capacity while maintaining a low self-weight. Under axial compression, the typical failure pattern was characterized by local steel plate bulging, localized crushing of the end concrete and overall flexural buckling about the weak axis.

(1) DCPEC columns exhibited favorable overall mechanical performance under axial compression. The plate elements of the two limbs developed local inward concavity at approximately one-third of the member height, while the flanges bulged outward and gradually lost their straightness. Local crushing was observed at the concrete near the column ends, whereas the concrete in the remaining regions remained largely intact. The global instability mode was dominated by weak-axis flexural buckling, indicating that the lightweight aggregate concrete infill not only enhanced the axial load-carrying capacity but also effectively delayed premature local instability of the cold-formed steel plates.

(2) The connection method and connection details had significant effects on the axial compressive resistance. Under identical conditions, the ultimate load of the bolted short column was approximately 8.4% higher than that of the welded short column. For the bare steel comparison specimens, the ultimate load of specimen KL-1 reached 282 kN, which was about 16% higher than that of specimen KH-1 (243 kN) and the welded specimen was more prone to develop torsion-coupled instability after the onset of local buckling. In the bolted specimens, increasing the number of connecting plates from 3 to 5 increased the ultimate load by about 40%. In the welded specimens, increasing the number of weld spots from 3 to 6 improved the ultimate load by approximately 10.4%, whereas a further increase from 6 to 11 led to an additional improvement of only about 6.7%, indicating a clear reduction in marginal benefit once the connection density became sufficient to ensure effective force transfer.

(3) Steel wall thickness and slenderness ratio were identified as the key parameters governing the axial performance of DCPEC columns. Increasing the wall thickness from 2.0 mm to 3.0 mm led to an average increase of about 16% in ultimate load. By contrast, increasing the slenderness ratio from 25.98 to 41.57 resulted in a reduction of about 30% in ultimate load. The finite element parametric analysis further indicated that when the slenderness ratio increased from 17.32 to 34.64, the ultimate load decreased by about 11.56%. These results confirm that excessive slenderness significantly impairs the axial resistance, whereas a larger wall thickness contributes to improved local stability and higher load-carrying capacity.

(4) The lightweight aggregate concrete infill exhibited a clear delaying effect on local buckling and distortional buckling. This effect is mainly attributed to the bidirectional composite mechanism in which the steel plates confine the lateral deformation of the concrete, while the concrete, in turn, provides internal support to the web and flanges through interfacial contact and friction. In the pure steel specimen KL-1, local bulging of the steel web occurred at about 70% of the ultimate load, whereas in the composite short column DL-1, steel plate bulging did not occur until approximately 90% of the ultimate load. This comparison demonstrates that the lightweight aggregate concrete effectively improved the local stability of the cold-formed steel plates.

(5) From an engineering perspective, DCPEC columns possess considerable potential for application in modular buildings, prefabricated light steel structures and other lightweight composite structural systems, owing to their advantages of low self-weight, relatively high bearing capacity and simple construction details. For the design of this type of member, in addition to local and global buckling of the steel plates, the additional restraint and support effect provided by the lightweight aggregate concrete infill should also be taken into account. Within the parameter range covered in this study, bolted connections are recommended as the preferred connection form; the steel wall thickness is recommended to be in the range of 2.5–3.0 mm; and the member slenderness ratio is recommended to be controlled within 17.32–25.98. These findings may provide useful references for the parameter selection, mechanism analysis and practical design of partially encased lightweight aggregate concrete composite columns composed of cold-formed thin-walled C-section steel.

## Figures and Tables

**Table 1:** Specimen design.

Specimen Number	Connection Methods	*Heights L* (mm)	Slenderness Ratio/λ	Number of Connecting Plates and Weld Spots	C-Section Steel Cross-Sectional/mm			
					Web Height/h (mm)	Flange Width/b (mm)	Lip Length/d (mm)	Steel Thickness/t (mm)
DL-1	bolted	1000	17.32	5	200	100	30	2.0
DL-2	bolted	1000	17.32	5	200	100	30	2.5
DL-3	bolted	1000	17.32	5	200	100	30	3.0
DL-4	bolted	1000	17.32	3	200	100	30	2.0
DH-1	welded	1000	17.32	6	200	100	30	2.0
DH-2	welded	1000	17.32	3	200	100	30	2.0
DH-3	welded	1000	17.32	11	200	100	30	2.0
DL-5	bolted	1500	25.98	7	200	100	30	2.0
DL-6	bolted	2000	34.64	9	200	100	30	2.0
DL-7	bolted	2400	41.57	11	200	100	30	2.0
KH-1	welded	1000	24.39	6	200	100	30	2.5
KL-1	bolted	1000	24.39	5	200	100	30	2.5

Note: “K” denotes the pure steel composite column without concrete infill, “D” represents the Double C-section partially encased composite (DCPEC) columns. “DL” indicates the DCPEC column with bolted connections, while “DH” denotes the DCPEC column with welded connections. “KL” indicates the pure steel column with bolted connections, while “KH” denotes the pure steel column with welded connections.

**Table 2:** LC25 concrete mix proportioning design.

Cement/kg/m^3^	Water/kg/m^3^	Sand/kg/m^3^	Fly Ash/kg/m^3^	Ceramsite/kg/m^3^	Water-Reducing/kg/m^3^
500	165	732	50	315	3

**Table 3:** Material performance test results.

Specimen Number	Steel Thickness/mm	Tensile Strength		Yield Strength		Elastic Modulus	
		Experimental Value/MPa	Mean Value/MPa	Experimental Value/MPa	Mean Value/MPa	Experimental Value/MPa	Mean Value/MPa
C-1	2.0	324	325	205	204	210,500	210,433
		318		198		209,800	
		332		210		211,000	
C-2	2.5	320	327	200	207	210,200	210,600
		328		208		210,700	
		335		215		210,900	
C-3	3.0	340	335	218	212	211,200	210,800
		337		212		210,800	
		330		206		210,400	

Note: Material mechanical property tests were conducted using a 100 kN microcomputer-controlled electronic universal testing machine to perform tensile tests on standard specimens. The testing procedure, methodology, specimen dimensions and machining accuracy strictly adhered to the provisions of the current national standard GB/T228.1-2021 “Metallic Materials-Tensile Testing-Part1: Method of Test at Room Temperature” [20].

**Table 4:** Specimen grouping and parameter-control scheme.

Specimen Group	Specimen Designation	Parameter Investigated Individually	Research Objective
Bare steel control group	KL-1, KH-1	Presence of lightweight aggregate concrete infill; connection type	To establish the axial compressive response of built-up double C-section steel columns without concrete infill, so as to quantify the contribution of lightweight aggregate concrete to load-carrying capacity enhancement and local stability improvement and to provide a benchmark for comparison of failure modes between composite and bare steel members.
Connection-type comparison group	DL-1, DH-1	Connection type	To compare the axial compressive behavior of short columns with bolted and welded built-up connections and to clarify the influence of connection details on the cooperative structural response and ultimate resistance of the member.
Wall thickness	DL-1, DL-2, DL-3	Steel thickness: 2.0, 2.5 and 3.0 mm	To investigate the effects of steel wall thickness and the corresponding width-to-thickness ratio on local stability, buckling evolution and axial compressive performance of the member.
Connecting-plate quantity comparison group	DL-1, DL-4	Number of connecting plates	To evaluate the influence of the arrangement density of connecting plates in bolted built-up columns on the cooperative action of the two C-section steels, the global stiffness and the axial load-carrying capacity.
Weld-spot quantity comparison group	DH-1, DH-2, DH-3	Number of weld spots/welding connection density	To investigate the effect of weld-spot quantity on sectional integrity, interfacial composite action and ultimate load-carrying capacity in welded built-up columns.
Slenderness-ratio comparison group	DL-5, DL-6, DL-7	The slenderness ratios were 25.98, 34.64 and 41.57	To investigate the influence of slenderness ratio on global stability, deformation development, failure mode and the degradation law of axial load-carrying capacity.

**Table 5:** Comparison of ultimate load between finite element simulation and experiment.

Specimen Number	Experiment/kN	Finite Element Simulation/kN	Relative Error
DL-1	1028	1050	2.14%
DL-2	1098	1140	3.82%
DL-3	1240	1320	6.45%
DL-4	725	786	8.41%
DH-1	1040	1129	8.55%
DH-2	942	1060	12.51%
DH-3	1110	1137	2.43%
DL-5	1200	1237	3.08%
DL-6	1080	1140	5.55%
DL-7	918	1029	12.09%

**Table 6:** Finite element analysis results of specimens with different wall thickness.

Specimen Number	Wall Thickness	Ultimate Load/kN
Y-1	1.50	1051
Y-2	2.00	1115
Y-3	2.50	1224
Y-4	3.00	1281
Y-6	1.50	1053
Y-7	2.00	1164
Y-8	2.50	1221
Y-9	3.00	1243
Y-12	1.50	1015
Y-13	2.00	1079
Y-14	2.50	1243
Y-15	3.00	1289

**Table 7:** Finite element analysis results of specimens with different slenderness ratios.

Specimen Number	Slenderness Ratio/λ	Ultimate Load/kN
Y-2	17.32	1220
Y-5	20.79	1185
Y-7	24.25	1160
Y-10	27.71	1134
Y-11	31.18	1105
Y-13	34.64	1079

## Data Availability

The original contributions presented in this study are included in the article. Further inquiries can be directed to the corresponding author.
